# Editorial: Optical interrogation of the nervous system: recent advances in optical techniques and their applications

**DOI:** 10.3389/fnins.2026.1916126

**Published:** 2026-09-22

**Authors:** Yuki Bando, Takashi Tominaga, Sachiko Tsuda

**Affiliations:** 1 Department of Anatomy and Neuroscience, Faculty of Medicine, Shimane University, Izumo, Japan; 2 Department of Organ and Tissue Anatomy, Hamamatsu University School of Medicine, Hamamatsu, Japan; 3 Institute of Neuroscience, Tokushima Bunri University, Sanuki, Japan; 4 Graduate School of Pharmaceutical Science, Tokushima Bunri University, Sanuki, Japan; 5 Kagawa School of Pharmaceutical Sciences, Tokushima Bunri University, Sanuki, Japan; 6 Division of Life Science, Graduate School of Science and Engineering, Saitama University, Saitama, Japan

**Keywords:** dendritic computation, imaging, neural circuits, neuropathology, non-linear optics, tissue clearing

The nervous system consists of a huge number of neurons and non-neuronal cells. To understand brain function, fine-scale analysis of both brain-wide structure and functions of neural circuits is essential. To achieve this, optical methods are useful. Recently, fluorescent probes for calcium, membrane potential, neurotransmitters and intracellular signals have been developed (Aggarwal et al., 2023; Bando et al., 2019, 2021; Yokoyama et al., 2024). The application of microbial opsins in neuroscience has enabled spatiotemporally precise manipulation of neuronal activity (Fenno et al., 2011). Furthermore, technical advances in microscopy have also contributed to neuroscience. The use of femtosecond pulse laser enabled multiphoton excitation which achieved high signal-to-noise ratio and deep penetration in scattering tissues (Denk et al., 1990). Combination of multiphoton microscopy and spatial light modulator allowed optical stimulation of neurons with desired patterns together with imaging neural activity (Yang et al., 2018). To track fast biological events, such as propagation of action potential, high- speed imaging is required. Employment of acousto-optic diffractor, microlens array or free-space angular-chirp-enhanced delay module has overcome limitation of the frame rate using conventional galvanometric and resonant scanners (Villette et al., 2019; Wu et al., 2020; Zhang et al., 2019). By applying these technologies, computations in dendrites and spines (Kwon et al., 2017), architecture of the functional neural circuits during development (Shiraishi et al., 2025), learning and plasticity (Makino and Komiyama, 2015) and pathological conditions have been intensely studied (Utsumi et al., 2023).

Optical methods have also been developed for investigation of brain-wide structures. Tissue clearing methods were developed for brain-wide imaging of cellular architectures of the brain (Susaki et al., 2014). Light sheet microscopy enables fine-scale imaging of brain structure at high speed (Susaki et al., 2014). Using these techniques, the whole architecture of the brain vascular system was described that was not possible with conventional confocal imaging of the brain sections (Kirst et al., 2020).

This Research Topic explores how optical methods enhance neuroscience research including development of novel techniques and application of optical methods to biological questions. This Research Topic consists of two original research articles, one methods article and one mini review article, that are summarized below.

Dendritic spines are small but fundamental computational compartments in neurons. Dendritic spines are small (~1 μm) protrusions that serve as the primary sites of excitatory synaptic inputs. Based on indirect measurements such as calcium imaging, it has long been debated whether spines are electrically compartmentalized (Yuste and Denk, 1995). To address this directly, Weng et al. applied two-photon holographic uncaging of caged glutamate using a spatial light modulator (SLM), synchronized with high-speed voltage imaging using an organic electrochromic dye. By directly measuring the uncaging-induced synaptic potentials using spatial averaging, taking advantage of the isopotential nature of these micro-compartments, they found that the signals were not attenuated across the spine neck. Furthermore, they revealed a distinct contrast in temporal summation: while inputs summate linearly at the soma, local summation within the spine is strictly sublinear, preventing synaptic saturation upon high-frequency uncaging. Together, these findings unveil the fundamental biophysical basis of computation in dendritic spines.

Advances in optical methods are critical not only for basic biological studies but also for clinical diagnosis. The intraoperative diagnosis of fibrous meningioma has been difficult, as conventional histopathology is time-consuming and alternative imaging methods lack sufficient spatial resolution. You et al. addressed this problem by developing a label-free, multimodal multiphoton microscopy (MPM) technique that uses second-harmonic generation (SHG) from collagen fibers and autofluorescence from NADH and FAD, along with a lambda mode for detailed spectral analysis. They successfully visualized the tumor microenvironment with high spatial resolution. Furthermore, they quantitatively analyzed the collagen content, demonstrating that this method could serve as a powerful tool for real-time, intraoperative diagnosis in neurosurgery.

To understand the pathology of neurodegenerative disorders in humans, circuit-wide analysis of brain structure is critical. However, the analysis of postmortem human brain tissue remains challenging due to autofluorescence, high lipid content, and long-term formaldehyde fixation. To overcome these hurdles, Rosen et al. developed SHARD (SHIELD, antigen retrieval, and delipidation), an improved method that combines antigen retrieval and tissue clearing. They also tested photobleaching before immunostaining, a widely used method to reduce autofluorescence in postmortem human tissues, but found that it did not significantly improve the signal-to-noise ratio. Furthermore, they provided a comprehensive guide to selecting optimal antibodies, successfully achieving highly multiplexed 3D immunofluorescence (up to seven colors) in long-term banked human brain tissues.

While fluorescence imaging is a powerful method, it has limitations, including photobleaching and potential alterations in cellular properties. For instance, transgenic mice expressing the fluorescent Ca^2+^ probe GCaMP6 have been reported to exhibit aberrant cortical activity (Steinmetz et al., 2017). Furthermore, conventional fluorophores are often too large to tag small bioactive molecules. To overcome these challenges, Nuriya highlights Coherent Raman Scattering (CRS) microscopy, including CARS (Coherent Anti-Stokes Raman Scattering) and SRS (Stimulated Raman Scattering), which significantly enhance the inherently weak Raman signals from vibrational modes of chemical bonds. A major breakthrough discussed is the use of “Raman tags”—such as alkyne or deuterium groups—which possess exceptionally small molecular weights and are uniquely identifiable in the “silent region” of the Raman spectrum. This innovation allows for the direct, target-specific visualization of previously inaccessible small molecules, including neurotransmitters, drugs, and water dynamics. Because Raman spectra are remarkably narrow, this approach also paves the way for highly multiplexed (over 10 colors) and quantitative chemical imaging, opening new frontiers in neuroscience research.

Overall, these articles provide significant advances in optical techniques for morphological and physiological studies in neuroscience. Collectively, they demonstrate how overcoming traditional limitations—whether in spatiotemporal resolution, tissue background, or molecular tagging—can drive breakthroughs across diverse fields. Ranging from fundamental synaptic physiology to the real-time clinical diagnosis of human brain tissues, the innovations presented in this Research Topic highlight the transformative power of optical methods. These ongoing developments will undoubtedly continue to illuminate the complex mechanisms of the nervous system.

## References

[B1] Aggarwal A. Liu R. Chen Y. Ralowicz A. J. Bergerson S. J. Tomaska F. . (2023). Glutamate indicators with improved activation kinetics and localization for imaging synaptic transmission. Nat. Methods 20, 925–934. doi: 10.1038/s41592-023-01863-6 PMC10250197 37142767

[B2] Bando Y. Grimm C. Cornejo V. H. Yuste R. (2019). Genetic voltage indicators. BMC Biol. 17, 71. doi: 10.1186/s12915-019-0682-0 31514747 PMC6739974

[B3] Bando Y. Wenzel M. Yuste R. (2021). Simultaneous two-photon imaging of action potentials and subthreshold inputs *in vivo*. Nat. Commun. 12, 7229. doi: 10.1038/s41467-021-27444-9 34893595 PMC8664861

[B4] Denk W. Strickler J. H Webb W. W. (1990). Two-photon laser scanning fluorescence microscopy. Science 248, 73–76. doi: 10.1126/science.2321027 2321027

[B5] Fenno L. Yizhar O. Deisseroth K. (2011). The development and application of optogenetics. Annu. Rev. Neurosci. 34, 389–412. doi: 10.1146/annurev-neuro-061010-113817 21692661 PMC6699620

[B6] Kirst C. Skriabine S. Vieites-Prado A. Topilko T. Bertin P. Gerschenfeld G. . (2020). Mapping the fine-scale organization and plasticity of the brain vasculature. Cell 180, 780–795.e25. doi: 10.1016/j.cell.2020.01.028 32059781

[B7] Kwon T. Sakamoto M. Peterka D. S. Yuste R. (2017). Attenuation of synaptic potentials in dendritic spines. Cell Rep. 20, 1100–1110. doi: 10.1016/j.celrep.2017.07.012 PMC5709047 28768195

[B8] Makino H. Komiyama T. (2015). Learning enhances the relative impact of top-down processing in the visual cortex. Nat. Neurosci. 18, 1116–1122. doi: 10.1038/nn.4061 PMC4523093 26167904

[B9] Shiraishi A. Hayashi A. Fukuda N. Hishinuma M. Miyazawa H. Tsuda S. (2025). Voltage imaging reveals the emergence of population activity in the spinal cord. eLife 14, 09641. doi: 10.7554/eLife.109641.1

[B10] Steinmetz N. A. Buetfering C. Lecoq J. Lee C. R. Peters A. J. Jacobs E. A. K. . (2017). Aberrant cortical activity in multiple GCaMP6-expressing transgenic mouse lines. eNeuro 4, ENEURO.0207-17.2017. doi: 10.1523/ENEURO.0207-17.2017 28932809 PMC5604087

[B11] Susaki E. A. Tainaka K. Perrin D. Kishino F. Tawara T. Watanabe T. M. . (2014). Whole-brain imaging with single-cell resolution using chemical cocktails and computational analysis. Cell 157, 726–739. doi: 10.1016/j.cell.2014.03.042 24746791

[B12] Utsumi Y. Taketoshi M. Miwa M. Tominaga Y. Tominaga T. (2023). Assessing seizure liability *in vitro* with voltage-sensitive dye imaging in mouse hippocampal slices. Front. Cell. Neurosci. 17, 1217368. doi: 10.3389/fncel.2023.1217368 PMC10481167 37680865

[B13] Villette V. Chavarha M. Dimov I. K. Bradley J. Pradhan L. Mathieu B. . (2019). Ultrafast two-photon imaging of a high-gain voltage indicator in awake behaving mice. Cell 179, 1590–1608.e23. doi: 10.1016/j.cell.2019.11.004 PMC6941988 31835034

[B14] Wu J. Liang Y. Chen S. Hsu C L. Chavarha M. Evans S W. . (2020). Kilohertz two-photon fluorescence microscopy imaging of neural activity *in vivo*. Nat. Methods 17, 287–290. doi: 10.1038/s41592-020-0762-7 PMC7199528 32123392

[B15] Yang W. Carrillo-Reid L. Bando Y. Peterka D. S. Yuste R. (2018). Simultaneous two-photon imaging and two-photon optogenetics of cortical circuits in three dimensions. eLife 7, e32671. doi: 10.7554/eLife.32671.019 PMC5832414 29412138

[B16] Yokoyama T. Manita S. Uwamori H. Tajiri M. Imayoshi I. Yagishita S. . (2024). A multicolor suite for deciphering population coding of calcium and cAMP *in vivo*. Nat. Methods 21, 897–907. doi: 10.1038/s41592-024-02222-9 38514778 PMC11093745

[B17] Yuste R. Denk W. (1995). Dendritic spines as basic functional units of neuronal integration. Nature 375, 682–684. doi: 10.1038/375682a0 7791901

[B18] Zhang T. Hernandez O. Chrapkiewicz R. Shai A. Wagner M. J. Zhang Y. . (2019). Kilohertz two-photon brain imaging in awake mice. Nat. Methods 16, 1119–1122. doi: 10.1038/s41592-019-0597-2 31659327 PMC9438750

